# Thematic Review of Serious Incidents in a Liaison Psychiatry Service

**DOI:** 10.1192/bjo.2024.416

**Published:** 2024-08-01

**Authors:** Imrana Puttaroo, Natasha Bunton, Michael Yousif, Aideen O'Halloran

**Affiliations:** West London NHS Trust, London, United Kingdom

## Abstract

**Aims:**

NHS England defines serious incidents as events in health care where the consequences are so significant that they warrant a comprehensive response. Serious incidents are individually reviewed, as per national standard practice, in our liaison psychiatry service line at West London NHS Trust. The aims of these individual reviews include system wide learning, organizational accountability and to make changes to the system to prevent a repetition.

There is currently no mandated requirement for thematic review of incidents. Therefore, there is a risk that long-term learning may be limited and overarching themes spanning the incidents may be missed. To improve this process, we have undertaken a thematic review of all serious incidents over a 2-year period, across the three teams in the liaison psychiatry service line.

The aims of this quality improvement project therefore were:

To understand persistent or recurrent systemic factors that contribute to serious incidents.

To identify priority areas for system changes in order to improve the safety of liaison psychiatry services.

To ensure lessons learnt from incidents are embedded within the liaison psychiatry service.

**Methods:**

This was a joint project undertaken by liaison psychiatry clinicians and the clinical governance team. Initially an inductive analysis of ten serious incidents took place. Over six months, we combed through the serious incident reports and collated the data. We then identified and stratified the key themes.

**Results:**

The 5 headline themes identified were:

1. Risk assessment and risk management.
2. Human factors.
3. Issues with referrals.
4. Triangle of care.
5. Organisational factors.

**Conclusion:**

The dominant theme which occurred across all cases was risk assessment and risk management. A narrow focus when considering risk and underestimation of risk led to the creation of suboptimal safety plans for patients. Our thematic analysis found a range of organisational factors, including the excessive demand on staff and resource limitations. Human factors are usually a reflection of organisational culture or system wide approaches. The issues we found with the implementation of the Triangle of Care reflect the need for a greater focus on involving families and carers.

The learning was shared with all staff in our annual development day, and this is planned to be an annual review of serious incidents across the liaison service. This approach should improve the depth of our learning and enable the service line to have an overview of the key themes which need to be addressed to deliver safer services.

